# Impact of an Eight-Week Plyometric Training Intervention on Neuromuscular Performance, Musculotendinous Stiffness, and Directional Speed in Elite Polish Badminton Athletes

**DOI:** 10.3390/jfmk10030304

**Published:** 2025-08-05

**Authors:** Mariola Gepfert, Artur Gołaś, Robert Roczniok, Jan Walencik, Kamil Węgrzynowicz, Adam Zając

**Affiliations:** 1 Institute of Sport Sciences, Academy of Physical Education, 40-065 Katowice, Poland; r.roczniok@awf.katowice.pl (R.R.); j.walencik@awf.katowice.pl (J.W.); k.wegrzynowicz@awf.katowice.pl (K.W.); a.zajac@awf.katowice.pl (A.Z.); 2 Wladyslaw Bieganski Collegium Medicum, Jan Dlugosz University in Czestochowa, 42-200 Czestochowa, Poland; a.golas@ujd.edu.pl

**Keywords:** plyometric training, badminton, speed, power, musculotendinous stiffness

## Abstract

**Background**: This study aimed to examine the effects of an 8-week plyometric training program on lower-limb explosive strength, jump performance, musculotendinous stiffness, reactive strength index (RSI), and multidirectional speed in elite Polish badminton players. **Methods**: Twenty-four athletes were randomly assigned to either an experimental group (n = 15), which supplemented their regular badminton training with plyometric exercises, or a control group (n = 15), which continued standard technical training. Performance assessments included squat jump (SJ), countermovement jump (CMJ), single-leg jumps, sprint tests (5 m, 10 m), lateral movements, musculotendinous stiffness, and RSI measurements. **Results**: The experimental group showed statistically significant improvements in jump height, power output, stiffness, and 10 m sprint and lateral slide-step performance (*p* < 0.05), with large effect sizes. No significant changes were observed in the control group. Single-leg jump improvements suggested potential benefits for addressing lower-limb asymmetries. **Conclusions**: An 8-week plyometric intervention significantly enhanced lower-limb explosive performance and multidirectional movement capabilities in young badminton players. These findings support the integration of targeted plyometric training into regular training programs to optimize physical performance, improve movement efficiency, and potentially reduce injury risk in high-intensity racket sports.

## 1. Introduction

Badminton is a dynamic high-intensity sport that requires a unique integration of agility, speed, strength, and precision [1]. Players must react instantaneously to shuttlecock velocities exceeding 300 km/h, necessitating rapid directional changes, quick reflexes, and powerful, yet accurate, strokes [2,3]. Effective court coverage is contingent upon explosive movement patterns initiated by a preparatory split-step, which facilitates swift transitions in all directions—forward, backward, lateral, and diagonal [4,5,6]. Proper footwork—comprising lunges, crossover steps, chasse steps, and small shuffles—minimizes energy expenditure, optimizes body positioning, and sustains movement efficiency throughout extended rallies [1,7]. Each stroke in badminton, including smashes, clears, drives, and drops, requires precise biomechanics and timing. In particular, high-intensity shots such as the smash demand maximal force output from the upper-limb kinetic chain while preserving accuracy [8,9]. Due to the game’s rapid pace, athletes must initiate new movement sequences almost instantaneously, leaving minimal transition time between offensive and defensive maneuvers. Electromyographic (EMG) analyses reveal significant activation of lower-limb muscles—especially the quadriceps, hamstrings, and gastrocnemius—during lunges and directional changes, underscoring the neuromuscular demands of badminton [10]. As in many individual and racket sports, repetitive unilateral loading of the dominant arm and leg contributes to muscular and coordination asymmetries, potentially increasing the risk of strength and endurance imbalances if not properly addressed [10,11]. Managing such asymmetries is therefore essential not only for maximizing performance but also for minimizing injury risk.

Musculotendinous stiffness, defined as the resistance of muscle–tendon units to elongation under load, plays a pivotal role in explosive actions by enabling efficient storage and rapid release of elastic energy [12]. Elevated stiffness around the ankle, knee, and hip joints enhances the reactive capability and supports seamless transitions between movements—factors critical for optimal footwork and recovery in badminton [13,14]. Adequate musculotendinous stiffness also facilitates balanced force distribution across limbs, thereby mitigating injury risk [15]. However, excessive stiffness may impair mobility and increase susceptibility to overuse or musculoskeletal injuries, underscoring the need for optimal regulation [16].

The Reactive Strength Index (RSI) a composite measure derived from the jump height and ground contact time—quantifies an athlete’s ability to rapidly switch from eccentric to concentric muscle action via the stretch-shortening cycle (SSC) [17,18]. Higher RSI values are associated with enhanced explosive strength, superior elastic energy utilization, improved court coverage, and more efficient performance during repeated high-intensity efforts [6,9,16]. Effective SSC engagement not only boosts movement reactivity but also reduces fatigue by limiting unnecessary energy expenditure [18]. Given the biomechanical and neuromuscular demands of badminton, training interventions aimed at improving RSI, musculotendinous stiffness, and lower-limb explosiveness are essential. Plyometric exercises, such as depth jumps, box jumps, and tuck jumps, have been shown to significantly enhance the reactive strength and elastic energy efficiency [19,20,21]. Additionally, sprint and agility drills that simulate on-court movement patterns further contribute to improvements in RSI and multidirectional speed [22].

Previous studies demonstrated that plyometric training significantly improves agility, sprint performance, and vertical jump height in badminton players [23,24,25,26]. These adaptations are critical for executing powerful strokes and maintaining court control during high-speed rallies. Furthermore, plyometric training has been shown to be effective in enhancing performance in agility tests (e.g., *T*-test, hexagon jump) and improving both unilateral and bilateral explosive power, with unilateral exercises showing particular benefits for single-leg jump performance and balance [27,28,29,30].

Therefore, the objective of the present study was to assess the effects of an eight-week plyometric training program on lower-limb explosive strength, reactive strength, musculotendinous stiffness, sprint performance, and functional asymmetry in elite Polish badminton players. It was hypothesized that targeted plyometric training would significantly improve the jump metrics, RSI, and multidirectional speed and reduce asymmetries, thereby contributing to enhanced athletic performance and injury prevention.

## 2. Materials and Methods

### 2.1. Participants

Thirty elite badminton players performing at the highest level of the game (18.8 ± 0.4 years, 171.3 ± 8.8 cm, 65.7 ± 8.2 kg and body fat 16.3 ± 4.2%) were randomly allocated to either the experimental (EG, n = 15) or the control training group (CG, n = 15). All participants underwent identical technical training in badminton throughout the duration of the study. Additionally, the ET group participated in an 8-week training program, which included two training sessions per week, each comprising 40 min of plyometric exercises. The CG did not perform additional plyometric training. All athletes are active members of the Academic Sports Association (AZS) in the Academy of Physical Education. In accordance with the regulations of the Polish Badminton Association, every participant had up-to-date medical clearance for competitive sport. All participants had a minimum of seven years of formal badminton training experience, ensuring a high level of technical proficiency and training maturity. Moreover, club-level athlete status and registration with AZS ensured the institutional consent for participation in the study.

The study protocol was approved by the Research Ethics Committee for Scientific Research at the Academy of Physical Education in Katowice, Poland (approval number: 3/2021), and was conducted in accordance with the Declaration of Helsinki (2013). All participants were informed about the procedures, benefits, and potential risks of the investigation, and they provided written informed consent prior to the start of the study. All data were anonymized and kept confidential, accessible only to the research team.

### 2.2. Testing Procedures

All participants reported to the testing site at a designated time to ensure standardized assessment conditions. Upon arrival, each athlete completed a general warm-up consisting of 5 min of low-intensity running, followed by dynamic stretching exercises targeting the lower limbs (e.g., leg swings, lunges, hip openers) and activation drills (such as high-knees, butt kicks, and skipping drills) to prepare for maximal-effort performance. A specific warm-up tailored to jumping and sprinting activities was subsequently performed, including submaximal jumps and accelerations.

#### 2.2.1. Sprint Test

**Figure 1:**
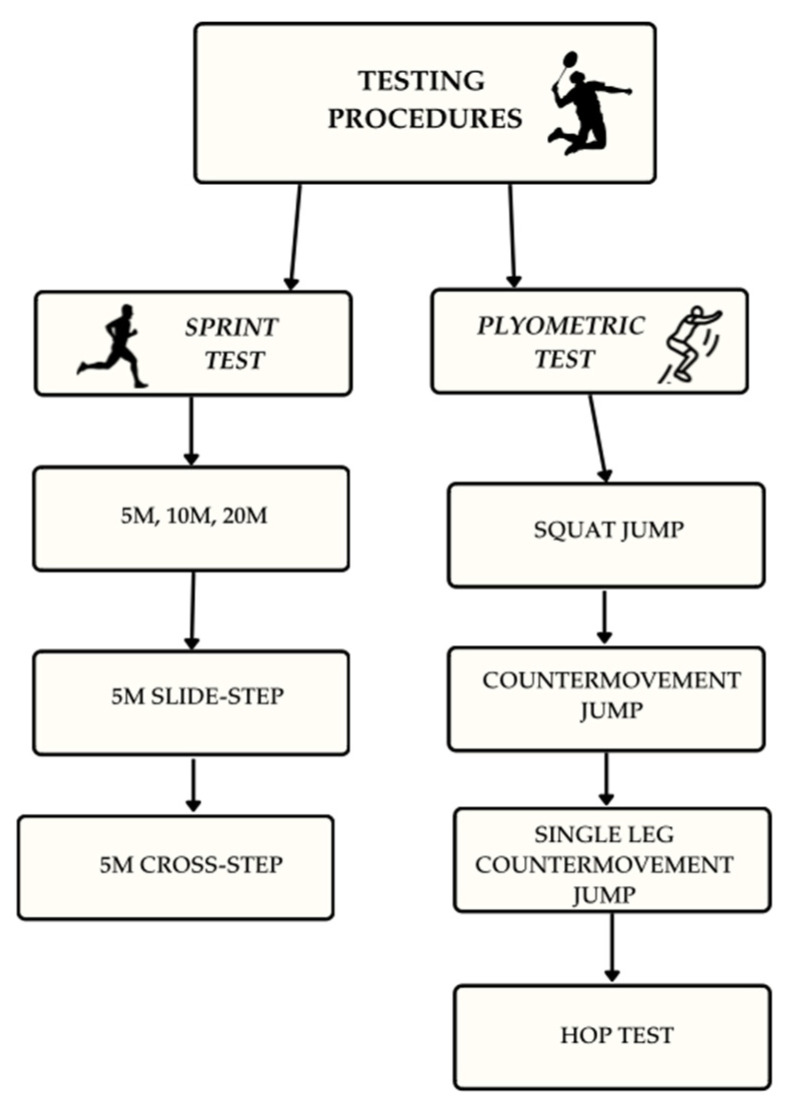
Testing procedures.

To maintain the uniformity of measurements, the tests were performed in a closed room (sports hall) using Microgate Photocells (Photocells Witty Gate, Bolzano, Italy) located at 5, 10, and 20 m. The athletes performed the following sprint tests: 5 m, 10 m, 20 m, 5 m slide-step to the right and left, 5 m cross-step to the right and left. Each subject performed two running tests, and the best time was selected for analysis (Figure 1).

#### 2.2.2. Analysis of Plyometric Parameters

All types of jumps were performed on the Chrono Jump platform (Chronojump Boscosystem, Barcelona, Spain), which demonstrates excellent reliability (intra-class correlation coefficient of 0.999–1.000). All athletes performed the ten Hop Test (HT) and three Squat Jump (SJ), Countermovement Jump (CMJ), and the Single Leg Jump (SLJ), with a 10 s rest interval between repetitions within each test. Additionally, a 3 min standardized rest period was implemented between different jump tests to minimize the risk of fatigue accumulation. The following variables were evaluated: jump height (based on the flight time measurement), peak power, RSI, and musculotendinous stiffness. The best attempt in terms of jump height was preserved for further analysis.

#### 2.2.3. Squat Jump, Countermovement Jump, Single Leg Jump, and Hop Test

The Squat Jump (SJ) is a type of vertical jump performed from a static squatting position without any countermovement. The athlete assumes a squatting position with knees bent at roughly 90 degrees and hands placed on the hips to prevent arm swing. From this static position, the athlete jumps vertically as high as possible using only leg power, without any preparatory countermovement. The athlete lands in a controlled manner, bending the knees to absorb impact.

The Countermovement Jump (CMJ) with arm swing has a starting position of a standing position with a straight torso and the knees fully extended, with the feet shoulder-width apart and the hands free to move. The participants were instructed to perform a quick downward movement (approximately 90° of knee flexion) and then a fast upward movement to jump as high as possible. Moreover, athletes were instructed to jump with both feet and knees fully extended (no leg tucking was allowed).

The Single Leg Countermovement jump (slCMJ) measures an athlete’s unilateral power, useful for sports that involve asymmetric or one-sided movements, such as badminton. All participants performed three SLs with swing arm and a 10as rest interval between each attempt. The slCMJ starting position was a one leg standing position with a straight torso and the one knee fully extended; the other leg was bent at 90°. The participants were instructed to perform a quick downward movement (approximately 90° of knee flexion) and then a fast upward movement to jump as high as possible. Moreover, athletes were instructed to jump with feet and knees fully extended (no leg tucking was allowed). The best attempt in terms of the jump height was preserved for further analysis.

The Hop Test (HT) is a dynamic test used to evaluate musculotendinous stiffness. It is particularly useful for assessing musculotendinous stiffness in the Achilles tendon and calf muscles, as well as overall lower-limb musculotendinous stiffness. The athlete performs a series of quick continuous hops in place. The goal is to minimize the ground contact time while maintaining a consistent height with each hop. Each landing should be controlled and with minimal “give” in the knees.

### 2.3. Training Procedures

The plyometric intervention program lasted 8 weeks and was conducted twice per week, with each session lasting approximately 40–45 min. The program was designed to progressively develop lower-limb explosive strength, landing mechanics, unilateral symmetry, and multidirectional power in badminton athletes. The entire progression was structured into four two-week phases, each increasing in complexity, intensity, and coordination demand.

**Figure 2:**
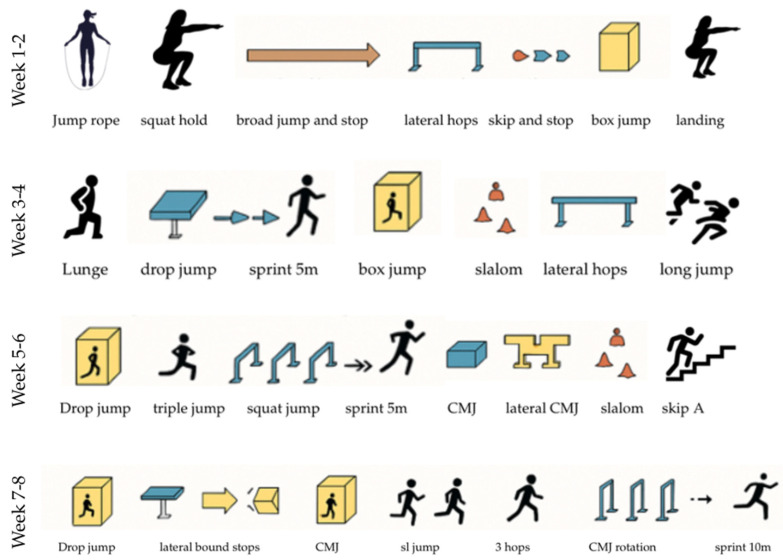
Visual representation of the 8-week plyometric training circuit used in the intervention program. Exercises progressed every two weeks in complexity, directionality, and intensity, focusing on bilateral and unilateral power development, landing control, and explosive movement patterns relevant to badminton performance.

As illustrated in Figure 2, the training circuit followed a station-based obstacle format. Each session included a warm-up, 5–7 plyometric tasks, and a cool-down phase. Athletes performed 3–4 full rounds of the course per session, with 60–90 s of rest between rounds.

#### Progressive Structure

Weeks 1–2: Technique and Landing Control

This segment focused on movement preparation and landing mechanics. Exercises included bodyweight squats, rope skipping, broad jump landings, lateral hops, skip-and-stop drills, and basic CMJ with soft landings. Emphasis was placed on joint alignment and deceleration control.

Weeks 3–4: Bilateral Explosiveness and COD Introduction

This segment introduced more dynamic movements such as drop jumps from platforms, linear sprints, directional slalom runs, and loaded countermovement jumps. Exercises began to integrate changes in direction and higher intensity jumps.

Weeks 5–6: Power Transfer and Unilateral Focus

This segment included triple jumps, unilateral bounding, elevated platform hops, and reactive landing drills. The complexity of movement patterns increased, requiring more neuromuscular coordination and lateral control.

Weeks 7–8: Integrated Power and Fatigue-Resistant Jumping

The final phase focused on high-load and fatigue-resistant jump tasks combining rotational elements (CMJ with 180° rotation), explosive sequences, and multidirectional movements (slalom + sprint, multiple low hurdle hops). These circuits mimicked competitive badminton footwork demands.

Each week introduced small modifications in volume (number of jumps or rounds), intensity (height or complexity), or direction (linear → lateral → multidirectional). The load was controlled through the obstacle height and task tempo. Emphasis was placed on quality of execution over quantity, and the athletes were monitored for technical fatigue.

This progression ensured safe adaptation to the neuromuscular load while enhancing specific performance factors required in elite badminton, including reactive power, postural control, and lower-limb asymmetry correction.

### 2.4. Statistical Methods

All analyses were performed using the Statistica v.13.1 package (StatSoft Polska, Kraków, Poland). Basic descriptive statistics were presented using means and standard deviations. Additionally, 95% confidence intervals for the mean values were calculated. The Shapiro–Wilk test, Levene’s test, and Mauchley’s test were applied, respectively, to verify the normality of distributions, homogeneity of variances, and sphericity assumptions. For verifying the significance of differences, a repeated measures MANOVA was used. Effect Sizes for main effects and interactions were determined with partial eta-squared (η^2^). Partial eta-squared values were classified as small (0.01 to 0.059), moderate (0.06 to 0.137), and large (>0.137). Post hoc comparisons for the parametric analysis of variance were conducted using Bonferroni’s test to identify which mean values differed significantly in the event of main effects or interactions. The significance level was set at α = 0.05. The G-Power software was used to estimate the a priori test power. Manova repeated measures, within-between interaction for two groups and two measurement series, with an effect size of at least 0.3, α = 0.05, and 1 − β = 0.95 and a correlation among repeated measures of 0.65, yielded a statistical power of 0.95 with a minimum sample size of 28 participants. For two groups and four measurement series, with an effect size of at least 0.3, α = 0.05, and 1 − β = 0.95 and a correlation among repeated measures above 0.5, a statistical power of 0.96 was achieved with a minimum sample size of 26 participants.

## 3. Results

### 3.1. 5 m Sprint, Slide-Step 5 m Left and Right, and Cross-Step 5 m Left and Right

**Figure 3:**
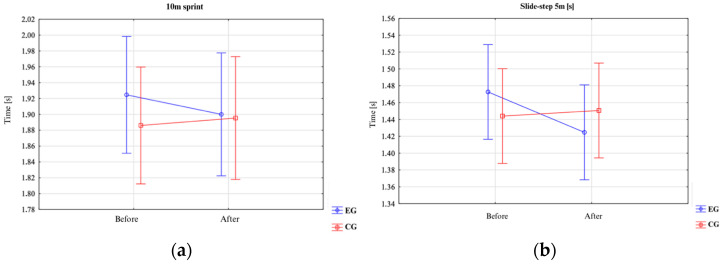
Differences between groups (EG vs. CG): (**a**) 10 m [s] sprint; (**b**) lateral 5 m [s] left.

**Figure 4:**
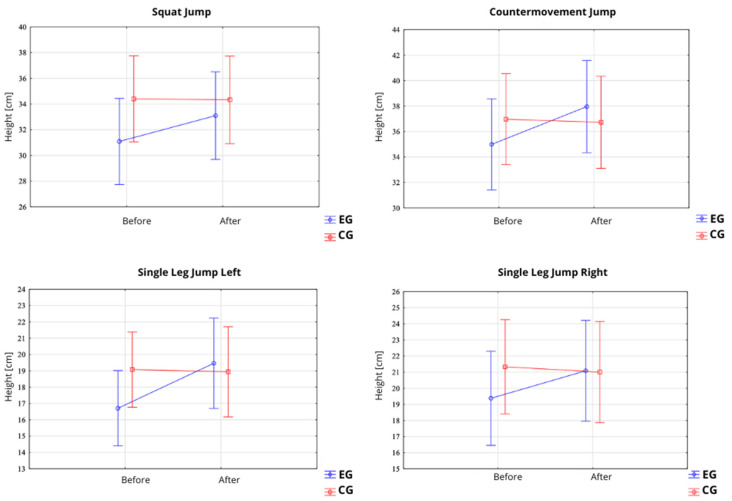
Between-group comparison of changes in height [cm] for squat jump, countermovement jump, and single-leg jump (**left** and **right** leg) measured pre- and post-intervention in the experimental (EG) and control (CG) groups.

An analysis of the results presented in Table 1 indicated that for the variables 5 m Sprint [s], Slide-Step 5 m [s] Right, Cross-Step 5 m [s] Left, and Cross-Step 5 m [s] Right, there were no statistically significant differences for the main effects of group (*p* > 0.05) or pre–post (*p* > 0.05), nor for the Group × pre–post interaction (*p* > 0.05). For the 10 m Sprint [s] variable, no statistically significant differences were observed for the main effects of group (*p* > 0.05) or pre–post (*p* > 0.05) (Figure 3). However, a significant difference emerged for the Group × pre–post interaction (F = 4.57; *p* = 0.041; η^2^ = 0.13). Specifically, the experimental group showed a statistically significant improvement in 10 m sprint time (*p* = 0.048). No significant differences were found in the control group (*p* = 0.44), and there were no differences between the groups prior to the experiment (*p* = 0.46). For the Slide-Step 5 m [s] Left variable, no statistically significant differences were observed for the main effects of group (*p* > 0.05) or pre–post (*p* > 0.05). However, there was a significant difference for the Group × pre–post interaction (F = 6.30; *p* = 0.018; η^2^ = 0.18). Specifically, the experimental group demonstrated a statistically significant improvement in the Slide-Step 5 m [s] Left performance time (*p* = 0.021) (Figure 4). No significant differences were noted in the control group (*p* = 0.97), and no differences were observed between the groups prior to the experiment (*p* = 0.88). These results are supported by the graphical interpretations.

#### 3.1.1. Squat Jump, Countermovement Jump, Single Leg CMJ Left and Right

**Figure 5:**
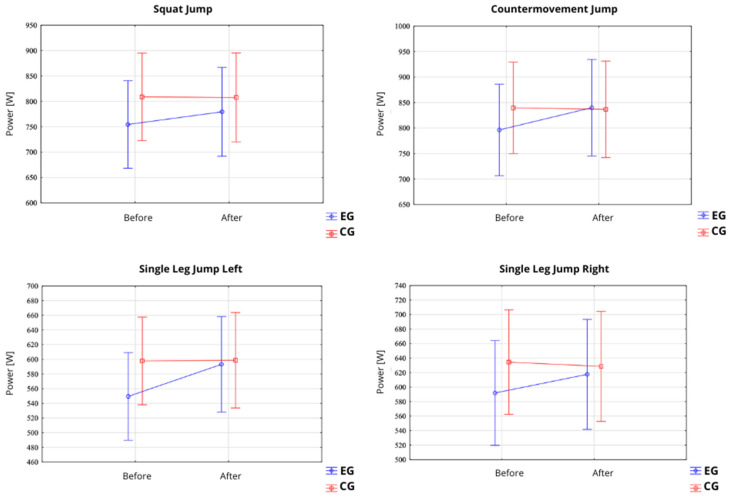
Between-group comparison of changes in power output [W] for squat jump, countermovement jump, and single-leg jump (**left** and **right** leg) measured pre- and post-intervention in the experimental (EG) and control (CG) groups.

In the SJ test, a significant main effect of time (pre–post) was observed for the jump height (F = 21.68; *p* = 0.0001; η^2^ = 0.44) (Table 2; Figure 4). In the EG, the jump height increased from 31.09 ± 6.73 cm (95% CI: 27.36–34.81) before the intervention to 33.10 ± 6.84 cm (95% CI: 29.31–36.88) after the intervention. In contrast, no significant changes were observed in the CG, where the jump height remained stable (34.40 ± 5.93 cm before vs. 34.33 ± 6.03 cm after the intervention). Similarly, a significant main effect of time was found for power output (F = 25.59; *p* = 0.0001; η^2^ = 0.42) (Figure 5). In the EG, the power output increased from 754.46 ± 164.20 W (95% CI: 663.53–845.39) to 779.37 ± 168.48 W (95% CI: 686.07–872.68). No significant changes were observed in the CG (808.78 ± 162.16 W before vs. 807.75 ± 162.46 W after the intervention). Moreover, a statistically significant interaction between group and time (Group × Pre–Post) was observed for both jump height (F = 25.08; *p* < 0.0001; η^2^ = 0.47) and power output (F = 24.31; *p* < 0.0001; η^2^ = 0.46), indicating that improvements occurred exclusively in the EG.

In the CMJ test, a significant main effect of time was also recorded for the jump height (F = 24.32; *p* < 0.0001; η^2^ = 0.46) (Figure 4). Participants in the EG improved their jump height from 35.00 ± 7.07 cm (95% CI: 31.08–38.91) to 37.96 ± 7.42 cm (95% CI: 33.85–42.07) after the intervention, whereas the CG did not show significant changes (36.97 ± 6.43 cm before vs. 36.72 ± 6.20 cm after). A significant main effect of time was likewise noted for power output (F = 10.87; *p* = 0.0027; η^2^ = 0.28) (Figure 5). In the EG, the power output increased from 796.21 ± 164.74 W (95% CI: 704.98–887.44) to 839.89 ± 185.31 W (95% CI: 737.27–942.51). Again, no significant differences were observed in the CG (839.51 ± 173.96 W before vs. 836.70 ± 172.17 W after the intervention). Statistically significant group × time interactions were observed for both the jump height (F = 34.00; *p* < 0.0001; η^2^ = 0.55) and power output (F = 14.06; *p* = 0.0008; η^2^ = 0.33), confirming that the improvements were specific to the EG.

Analysis of the overall results indicated that for all examined variables, the main effect of group was not statistically significant (*p* > 0.05). However, the main effect of time (pre–post) was statistically significant for all variables (*p* < 0.05). Additionally, all group × pre–post interactions were statistically significant, confirming that the observed changes were specific to the experimental group and not to the control group.

In the slCMJleft test, a significant main effect of time (pre–post) was observed for jump height (F = 15.94; *p* = 0.0004; η^2^ = 0.36). In the EG, the jump height increased from 16.70 ± 4.60 cm (95% CI: 14.16–19.25) before the intervention to 19.46 ± 5.55 cm (95% CI: 16.39–22.54) after the intervention (Table 3; Figure 4). No significant change was noted in the CG, where the jump height remained relatively stable (19.08 ± 4.13 cm before vs. 18.94 ± 4.89 cm after the intervention). Regarding power output, a significant main effect of time was also recorded (F = 15.86; *p* = 0.0004; η^2^ = 0.36).

In the EG, the power output increased from 549.51 ± 116.93 W (95% CI: 484.76–614.26) to 593.21 ± 128.46 W (95% CI: 522.07–664.35), whereas the CG showed no notable changes (597.82 ± 108.98 W before vs. 598.78 ± 117.45 W after). A statistically significant group × time interaction was observed for both the jump height (F = 19.40; *p* = 0.0001; η^2^ = 0.41) and power output (F = 14.53; *p* = 0.0007; η^2^ = 0.34), indicating that improvements occurred exclusively in the experimental group (Table 3, Figure 5).

**Figure 6:**
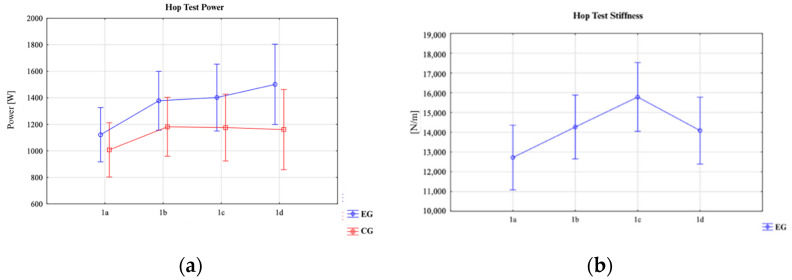
Hop test (**a**) power [W] changes after repeated trials in EG and CG (pre-intervention); (**b**) musculotendinous stiffness [N/m] in the hop test for both groups.

In the slCMJright test, a significant main effect of time was also observed for the jump height (F = 4.86; *p* = 0.036; η^2^ = 0.15). In the EG, the jump height improved from 19.38 ± 5.82 cm (95% CI: 16.15–22.60) to 21.09 ± 6.04 cm (95% CI: 17.74–24.43) following the intervention (Figure 5,6). No significant change was detected in the CG (21.33 ± 5.22 cm before vs. 21.01 ± 5.81 cm after the intervention). For power output, a trend toward significance for the main effect of time was observed (F = 3.91; *p* = 0.058; η^2^ = 0.12), while the group × time interaction was statistically significant (F = 9.89; *p* = 0.0039; η^2^ = 0.26). The EG demonstrated an increase in power from 591.88 ± 138.11 W (95% CI: 515.39–668.36) to 617.59 ± 144.44 W (95% CI: 537.60–697.58), while the CG exhibited no meaningful changes (634.40 ± 134.56 W before vs. 628.54 ± 142.24 W after).

#### 3.1.2. Hop Test

An analysis of the data presented in Table 4 revealed no statistically significant main effects or group × time interactions for the variables jump height and RSI (*p* > 0.05). In terms of jump height, the EG demonstrated relatively stable values across all measurements, ranging from 26.74 ± 3.06 cm (95% CI: 24.97–28.51) at 1a to 36.17 ± 35.89 cm (95% CI: 15.45–56.90) at 1d. Similarly, the CG presented comparable results, with values ranging from 24.31 ± 7.72 cm (95% CI: 19.85–28.76) to 29.16 ± 15.12 cm (95% CI: 20.43–37.89). For the RSI, the EG exhibited an increase from 1.06 ± 0.20 cm/s (95% CI: 0.95–1.18) at 1a to 1.46 ± 1.05 cm/s (95% CI: 0.85–2.06) at 1d, while the CG ranged from 0.99 ± 0.43 cm/s (95% CI: 0.74–1.23) to 1.33 ± 0.87 cm/s (95% CI: 0.82–1.83). However, these changes were not statistically significant. In contrast, significant differences were observed in power output and lower-limb musculotendinous stiffness. A significant main effect of time was found for power output (F = 3.79; *p* = 0.01; η^2^ = 0.13).

In the EG, the power output increased from 1121.54 ± 385.88 W (95% CI: 898.74–1344.34) at 1a to 1501.20 ± 660.08 W (95% CI: 1120.08–1882.32) at 1d. Similarly, the CG showed an increase from 1007.70 ± 358.86 W (95% CI: 800.50–1214.90) at 1a to 1160.54 ± 414.02 W (95% CI: 921.49–1399.59) at 1d, although this increase was not statistically significant in the group × time interaction (Figure 6).

The most pronounced changes were observed in lower-limb musculotendinous stiffness. A highly significant main effect of time was found (F = 10.46; *p* < 0.0001; η^2^ = 0.29). The EG improved from 13,605.30 ± 4392.73 N/m (95% CI: 11,069.01–16,141.59) at 1a to 15,365.27 ± 3955.07 N/m (95% CI: 13,081.68–17,648.86) at 1d, peaking at 16,910.75 ± 4940.17 N/m (95% CI: 14,058.38–19,763.12) at 1c. The CG showed a similar trend, increasing from 11,838.15 ± 4027.11 N/m (95% CI: 9512.97–14,163.34) at 1a to 12,804.93 ± 4716.03 N/m (95% CI: 10,081.97–15,527.88) at 1d. Post-hoc analysis confirmed that musculotendinous stiffness significantly increased at time point 1b compared to 1a (*p* = 0.037), at 1c compared to 1a (*p* < 0.0001), at 1c compared to 1b (*p* = 0.042), and at 1c compared to 1d (*p* = 0.016). While no significant changes were observed for the jump height and RSI, the intervention led to significant improvements in the lower-limb power output and musculotendinous stiffness over time, particularly in the experimental group.

### 3.2. Formatting of Mathematical Components

In order to analyze the effects of the eight-week plyometric training program, we applied a repeated-measures analysis of variance (ANOVA) and multivariate analysis of variance (MANOVA) models. The analyses were performed with a significance level set at α = 0.05.

#### 3.2.1. Repeated Measures ANOVA Model

The statistical model used to evaluate the main effects and interactions is represented as

Y_klm_ = μ + α_k_ + β_l_ + (αβ)_kl_ + ε_klm_, (1)

where Y_klm_ is the observed value for the i-th group and j-th time point, μ is the overall mean, α_k_ is the fixed effect of group (experimental vs. control), β_l_ is the fixed effect of time (pre vs. post), (αβ)_kl_ represents the interaction between group and time, and ε_klm_ is the random error term.

#### 3.2.2. Effect Size (Partial Eta-Squared)

Effect sizes for the ANOVA were calculated using partial eta-squared (η^2^), defined as

η^2^ = SS_exao_/(SS_exao_ + SS_haxxao_), (2)

where SS_exao_ is the sum of squares for the factor, and SS_haxxao_ is the sum of squares for the error.

#### 3.2.3. A Priori Power Analysis

Sample size adequacy was verified using G*Power software version 3.1.9.7. The formula for estimating the required sample size N is

N = [(Z_1−_β + Z_1−_α)^2^…2σ^2^]/(μ_1_ − μ_2_)^2^, (3)

where Z_1−_β and Z_1−_α represent the z-scores corresponding to power and alpha levels respectively, σ^2^ is the estimated variance, and (μ_1_ − μ_2_) is the anticipated mean difference.

#### 3.2.4. Reactive Strength Index (RSI)

The reactive strength index (RSI) was calculated as

RSI = Jump Height (cm)/Ground Contact Time (s).(4)

Higher RSI values reflect better utilization of the stretch-shortening cycle (SSC) and improved neuromuscular reactivity.

#### 3.2.5. Theorem-Type Statement

**Theorem** **1.** *A structured eight-week plyometric training intervention significantly increases lower-limb power output and musculotendinous stiffness in elite badminton players*.

**Proof** **of** **Theorem** **1.** Statistical analysis revealed significant improvements in power (F = 3.79; *p* = 0.01; η^2^ = 0.13) and musculotendinous stiffness (F = 10.46; *p* < 0.0001; η^2^ = 0.29) following the intervention, with changes observed exclusively in the experimental group. These findings validate the hypothesis. □

Next, we provide further interpretation and discussion of the findings.

## 4. Discussion

The primary objective of the present study was to examine the effects of an eight-week plyometric training program on the jump performance, power output, reactive strength, musculotendinous stiffness, and multidirectional speed in elite badminton players. The findings revealed significant enhancements in lower-limb explosive capabilities—particularly in the jump height, power output, musculotendinous stiffness, and selected sprint and agility measures—in the experimental group (EG), with no notable changes observed in the control group (CG).

These results are consistent with the previous literature demonstrating the efficacy of plyometric training in improving neuromuscular performance through enhanced utilization of the stretch-shortening cycle (SSC), increased motor unit recruitment, improved intermuscular coordination, and more effective elastic energy storage [31,32,33]. Plyometric training induces both muscular and neural adaptations, which likely underlie the observed improvements in rapid force generation and vertical jump performance. In the context of badminton, the ability to produce explosive lower-limb power is essential for executing high-intensity actions such as jump smashes, dynamic lunges, and rapid directional shifts [33].

The significant improvements in the Squat Jump (SJ) and Countermovement Jump (CMJ) performance observed in the EG are consistent with prior work demonstrating the positive effects of plyometric training on the jump height, RSI, and movement economy in athletes [34,35]. In addition to improvements in vertical jump, significant enhancements were observed in the 10 m sprint and the 5 m lateral slide-step to the left. These findings are consistent with previous research indicating that plyometric training enhances short-distance sprinting and lateral agility—key attributes for efficient court coverage in badminton [23,36]. The absence of improvement in the 5 m linear sprint and crossover steps suggests that the applied training protocol was more effective for enhancing acceleration and lateral movement over slightly longer distances. Future research should explore the design of sport-specific plyometric progressions tailored to target very short multidirectional movement tasks. Of particular interest is the asymmetric improvement observed in the lateral slide-step test, where significant enhancement occurred only for movement to the left side. This asymmetry may reflect habitual dominance in limb usage, a common feature in racquet sports such as badminton. Players often exhibit functional asymmetries in strength, coordination, and movement control due to repetitive use of one limb [10]. The training intervention may have contributed to reducing this imbalance by improving force production or neuromuscular control in the non-dominant limb. This finding highlights the potential of plyometric training not only to enhance performance but also to act as a corrective mechanism for addressing inter-limb asymmetries. Improvements in single-leg jump performance, particularly on the left side, further underscore the value of plyometric interventions in correcting unilateral deficits. This is especially relevant in sports characterized by repetitive unilateral loading, where asymmetries may predispose athletes to overuse injuries. Prior studies support the efficacy of both bilateral and unilateral plyometric exercises in enhancing single-leg function, with unilateral modalities conferring additional benefits for dynamic stability and limb-specific adaptations [29,37,38].

Although the gains in musculotendinous stiffness and power output were statistically significant, no improvements were observed in the RSI during repeated hop tests. This could be attributed to the relatively short duration of the training program or the moderate intensity of the SSC stimulus. RSI is known to be highly sensitive to the duration, intensity, and specificity of training and may require longer exposure or higher loading thresholds to elicit significant adaptations [13]. Furthermore, individual baseline RSI values and inter-individual differences in neuromuscular efficiency could influence responsiveness to the training intervention. Future protocols may consider incorporating higher-intensity drop jumps or extending the intervention beyond eight weeks to more effectively target RSI.

Nevertheless, the observed increases in musculotendinous stiffness support the role of plyometric training in enhancing elastic energy reutilization—an essential attribute for efficient movement during high-intensity rally play [38,39,40]. These physiological adaptations contribute to improved explosive performance, agility, and endurance, thereby justifying the inclusion of plyometric training in comprehensive athletic development programs [39,40,41]. Importantly, while the present study primarily focused on biomechanical and neuromuscular markers, the observed adaptations bear direct relevance to competitive badminton performance. Increased vertical jump height may enhance smash reach and power, while improvements in lateral speed may lead to quicker positioning and superior defensive recovery. Elevated musculotendinous stiffness and reactive strength could also facilitate energy-efficient transitions between actions, thereby reducing fatigue and preserving performance during prolonged rallies.

A critical limitation of the current study, however, is the lack of direct sport-specific performance assessments—such as shuttlecock velocity, movement kinematics during match play, or rally duration. Although the neuromuscular adaptations observed are strongly associated with key performance actions in badminton, the absence of objective match-related data somewhat limits the practical applicability of the results. Future research should aim to integrate biomechanical, physiological, and technical–tactical assessments to better quantify the transfer of physical training adaptations to real-world competitive performance [42,43]. The lack of any meaningful improvements in the control group—despite the continuation of regular technical badminton training—underscores the insufficiency of technical and tactical training alone to elicit neuromuscular adaptations in youth athletes. This observation is consistent with prior reports highlighting the need for structured conditioning programs to support physical development in this population [44].

Although the present eight-week intervention elicited significant improvements in several key performance domains, previous studies suggest that longer interventions (e.g., 10–12 weeks) or individualized training programs may yield even greater gains [30,44,45,46]. Moreover, combining plyometric training with complementary modalities—such as sprint technique drills or resistance training—may further enhance transfer to sport-specific actions.

A notable strength of the present study lies in the use of objective and reliable biomechanical assessment tools to quantify jump performance, sprint ability, and musculotendinous stiffness. Nonetheless, limitations must be acknowledged. The relatively small sample size and short intervention duration may restrict the generalizability of the findings. Furthermore, the lack of sport-specific performance metrics limits the ecological validity of the results and should be addressed in future investigations to provide a more comprehensive understanding of training transfer to competition.

Moreover, a potential limitation of the present study lies in the absence of direct sport-specific performance metrics, such as shuttlecock velocity, movement patterns during match play, or rally duration. While the neuromuscular and biomechanical improvements observed—such as enhanced jump height, stiffness, and multidirectional speed—are strongly associated with badminton-specific actions (e.g., smashes, lunges, and directional changes), the lack of match-based performance data somewhat limits the translational applicability of our results. Future studies should aim to integrate technical-tactical analyses (e.g., video tracking, kinematic profiling, shuttle velocity) alongside biomechanical measures to provide a more complete picture of functional transfer. This approach will allow for a better understanding of how physical adaptations influence actual in-game effectiveness, decision-making speed, and recovery capacity during high-intensity rallies. Such multi-dimensional assessments will be crucial to validate the efficacy of plyometric interventions in elite badminton performance settings. In conclusion, this study demonstrates that an eight-week structured plyometric training program can significantly enhance the lower-limb explosive strength, vertical jump height, musculotendinous stiffness, and multidirectional movement performance in elite badminton players. The protocol also appears to offer corrective potential for addressing unilateral asymmetries. Integrating targeted plyometric exercises into standard training regimens represents a valuable strategy to optimize performance, improve movement efficiency, and potentially reduce injury risk in high-intensity racket sports.

## 5. Conclusions

The present study demonstrated that an eight-week plyometric training intervention significantly improved lower-limb explosive strength, vertical jump height, power output, musculotendinous stiffness, and selected multidirectional speed components in elite badminton players. The improvements observed exclusively in the experimental group highlight the effectiveness of integrating structured plyometric training into traditional sport-specific routines. These findings emphasize the importance of targeting neuromuscular development to enhance explosive athletic performance in dynamic high-speed sports such as badminton. The study reinforces the role of the stretch-shortening cycle (SSC) in facilitating explosive movements and confirms that lower-limb plyometric exercises effectively transfer to sport-specific movement skills critical for badminton performance. Despite the short intervention period, substantial performance improvements were recorded, suggesting that even relatively brief well-structured plyometric programs can meaningfully enhance key physical capacities in young athletes. The incorporation of such targeted interventions may not only optimize performance but also contribute to improved movement efficiency and reduced injury risk through better neuromuscular control and limb symmetry.

## Figures and Tables

**Table 1:** Descriptive statistics (M ± SD; 95% CI) and repeated measures ANOVA results for sprint and agility test times (5 m Sprint, 10 m Sprint, Slide-Step, Cross-Step—left and right) in experimental (EG) and control (CG) groups before and after the intervention.

Types	Group	M ± SD (−95%; 95% CI)		Main Effects and Interaction for MANOVA with Repeated Measures F/*p*/η^2^
		Before	After	
5 m Sprint [s]	EG	1.14 ± 0.11 (1.08; 1.19)	1.13 ± 0.15 (1.05; 1.21)	Group: F = 1.82; *p* = 0.19; η^2^ = 0.06 Before–After: F < 0.0001; *p* = 0.98; η^2^ < 0.001 Group Before–After: F = 0.25; *p* = 0.62; η^2^ = 0.001
	CG	1.08 ± 0.07 (1.04; 1.12)	1.09 ± 0.06 (1.05; 1.12)	
10 m Sprint [s]	EG	1.92 ± 0.16 * (1.84; 2.01)	1.90 ± 0.16 * (1.81; 1.99)	Group: F = 0.18; *p* = 0.68; η^2^ = 0.01 Before–After: F = 0.83; *p* = 0.37; η^2^ = 0.03 Group Before–After: F = 4.57; *p* = 0.041; η^2^ = 0.13
	CG	1.89 ± 0.12 (1.82; 1.95)	1.90 ± 0.13 (1.82; 1.97)	
Slide-Step 5 m [s] Left	EG	1.47 ± 0.11 * (1.41; 1.53)	1.42 ± 0.12 * (1.36; 1.49)	Group: F = 0.001; *p* = 0.97; η^2^ < 0.0001 Before–After: F = 3.60; *p* = 0.068; η^2^ = 0.11 Group Before–After: F = 6.30; *p* = 0.018; η^2^ = 0.18
	CG	1.44 ± 0.11 (1.39; 1.50)	1.45 ± 0.09 (1.40; 1.50)	
Slide-Step 5 m [s] Right	EG	1.46 ± 0.13 (1.39; 1.53)	1.43 ± 0.13 (1.36; 1.50)	Group: F = 0.95; *p* = 0.34; η^2^ = 0.033 Before–After: F = 1.79; *p* = 0.19; η^2^ = 0.060 Group Before–After: F = 0.55; *p* = 0.46; η^2^ = 0.019
	CG	1.49 ± 0.13 (1.42; 1.56)	1.48 ± 0.11 (1.42; 1.54)	
Cross-Step 5 m [s] Left	EG	1.28 ± 0.10 (1.22; 1.33)	1.26 ± 0.11 (1.19; 1.32)	Group: F = 3.00; *p* = 0.094; η^2^ = 0.097 Before–After: F = 1.46; *p* = 0.24; η^2^ = 0.050 Group Before–After: F = 1.66; *p* = 0.21; η^2^ = 0.056
	CG	1.33 ± 0.09 (1.28; 1.37)	1.33 ± 0.09 (1.28; 1.38)	
Cross-Step 5 m [s] Right	EG	1.26 ± 0.10 (1.20; 1.32)	1.25 ± 0.14 (1.17; 1.33)	Group: F = 3.95; *p* = 0.06; η^2^ = 0.12 Before–After: F = 0.06; *p* = 0.81; η^2^ = 0.002 Group Before–After: F = 0.03; *p* = 0.86; η^2^ = 0.001
	CG	1.34 ± 0.11 (1.28; 1.40)	1.34 ± 0.10 (1.28; 1.39)	

* *p* < 0.05.

**Table 2:** Descriptive statistics (M ± SD) and repeated measures ANOVA results for SJ and CMJ height and power in the experimental (EG) and control (CG) groups before and after the intervention.

Types	Parameters	G	Before	After	Main Effects and Interaction for MANOVA with Repeated Measures F/*p*/η^2^
			M ± SD (−95%; 95% CI)	M ± SD (−95%; 95% CI)	
SJ	Height [cm]	EG	31.09 ± 6.73 *** (27.36; 34.81)	33.10 ± 6.84 *** (29.31; 36.88)	Group: F = 0.95; *p* = 0.34; η^2^ = 0.029 Before–After: F = 21.68; *p* = 0.0001; η^2^ = 0.44 Group Before–After: F = 25.08; *p* < 0.0001; η^2^ = 0.47
		CG	34.40 ± 5.93 (31.12; 37.68)	34.33 ± 6.03 (30.99; 37.67)	
	Power [W]	EG	754.46 ± 164.20 *** (663.53; 845.39)	779.37 ± 168.48 *** (686.07; 872.68)	Group: F = 0.48; *p* = 0.50; η^2^ = 0.020 Before–After: F = 25.59; *p* = 0.0001; η^2^ = 0.42 Group Before–After: F = 24.31; *p* < 0.0001; η^2^ = 0.46
		CG	808.78 ± 162.16 (718.98; 898.58)	807.75 ± 162.46 (717.78; 897.72)	
CMJ	Height [cm]	EG	35.00 ± 7.07 *** (31.08; 38.91)	37.96 ± 7.42 *** (33.85; 42.07)	Group: F = 0.02; *p* = 0.88; η^2^ = 0.001 Before–After: F = 24.32; *p* < 0.0001; η^2^ = 0.46 Group Before–After: F = 34.00; *p* < 0.0001; η^2^ = 0.55
		CG	36.97 ± 6.43 (33.41; 40.53)	36.72 ± 6.20 (33.29; 40.16)	
	Power [W]	EG	796.21 ± 164.74 *** (704.98; 887.44)	839.89 ± 185.31 *** (737.27; 942.51)	Group: F = 0.10; *p* = 0.75; η^2^ = 0.004 Before–After: F = 10.87; *p* = 0.0027; η^2^ = 0.28 Group Before–After: F = 14.06; *p* = 0.0008; η^2^ = 0.33
		CG	839.51 ± 173.96 (743.17; 935.84)	836.70 ± 172.17 (741.36; 932.05)	

*** *p* < 0.001.

**Table 3:** Descriptive statistics (M ± SD) and repeated measures ANOVA results for single-leg countermovement jump (slCMJ) height and power (left and right leg) in the experimental (EG) and control (CG) groups before and after the intervention.

Types	Parameters	G	Before	After	Main Effects and Interaction for MANOVA with Repeated Measures F/*p*/η^2^
			M ± SD (−95%; 95% CI)	M ± SD (−95%; 95% CI)	
slCMJleft	Height [cm]	EG	16.70 ± 4.60 *** (14.16; 19.25)	19.46 ± 5.55 *** (16.39; 22.54)	Group: F = 0.29; *p* = 0.60; η^2^ = 0.099 Before–After: F = 15.94; *p* = 0.0004; η^2^ = 0.36 Group Before–After: F = 19.40; *p* = 0.0001; η^2^ = 0.41
		CG	19.08 ± 4.13 (16.79; 21.36)	18.94 ± 4.89 (16.23; 21.65)	
	Power [W]	EG	549.51 ± 116.93 *** (484.76; 614.26)	593.21 ± 128.46 *** (522.07; 664.35)	Group: F = 0.40; *p* = 0.53; η^2^ = 0.01 Before–After: F = 15.86; *p* = 0.0004; η^2^ = 0.36 Group Before–After: F = 14.53; *p* = 0.0007; η^2^ = 0.34
		CG	597.82 ± 108.98 (537.47; 658.18)	598.78 ± 117.45 (533.73; 663.82)	
slCMJright	Height [cm]	EG	19.38 ± 5.82 ** (16.15; 22.60)	21.09 ± 6.04 ** (17.74; 24.43)	Group: F = 0.21; *p* = 0.65; η^2^ = 0.007 Before–After: F = 4.86; *p* = 0.036; η^2^ = 0.15 Group Before–After: F = 10.60; *p* = 0.0030; η^2^ = 0.27
		CG	21.33 ± 5.22 (18.44; 24.23)	21.01 ± 5.81 (17.79; 24.22)	
	Power [W]	EG	591.88 ± 138.11 ** (515.39; 668.36)	617.59 ± 144.44 ** (537.60; 697.58)	Group: F = 0.28; *p* = 0.60; η^2^ = 0.01 Before–After: F = 3.91; *p* = 0.058; η^2^ = 0.12 Group Before–After: F = 9.89; *p* = 0.0039; η^2^ = 0.26
		CG	634.40 ± 134.56 (559.89; 708.92)	628.54 ± 142.24 (549.77; 707.31)	

** *p* < 0.01; *** *p* < 0.001.

**Table 4:** Descriptive statistics (M ± SD) and repeated measures ANOVA results for hop test variables (jump height, RSI, power) across three trials (1a–1c) in the experimental (EG) and control (CG) groups prior to training intervention.

Parameters		Group		Main Effects and Interaction for MANOVA with Repeated Measures F/*p*/η^2^
		EG	CG	
		M ± SD (95%; 95% CI)	M ± SD (95%; 95% CI)	
Height [cm]	1a	26.74 ± 3.06 (24.97; 28.51)	24.31 ± 7.72 (19.85; 28.76)	Group: F = 0.70; *p* = 0.41; η^2^ = 0.026 Before–After: F = 1.05; *p* = 0.37; η^2^ = 0.039 Group Before–After: F = 0.85; *p* = 0.47; η^2^ = 0.031
	1b	26.58 ± 3.95 (24.30; 28.87)	29.16 ± 15.12 (20.43; 37.89)	
	1c	28.12 ± 4.16 (25.72; 30.53)	25.69 ± 9.39 (20.27; 31.12)	
	1d	36.17 ± 35.89 (15.45; 56.90)	27.20 ± 6.70 (23.34; 31.07)	
RSI [cm/s]	1a	1.06 ± 0.20 (0.95; 1.18)	0.99 ± 0.43 (0.74; 1.23)	Group: F = 0.43; *p* = 0.52; η^2^ = 0.016 Before–After: F = 1.75; *p* = 0.16; η^2^ = 0.063 Group Before–After: F = 0.71; *p* = 0.55; η^2^ = 0.027
	1b	1.21 ± 0.32 (1.02; 1.40)	1.33 ± 0.87 (0.82; 1.83)	
	1c	1.25 ± 0.30 (1.08; 1.43)	1.12 ± 0.54 (0.80; 1.43)	
	1d	1.46 ± 1.05 (0.85; 2.06)	1.18 ± 0.42 (0.94; 1.42)	
Power [W]	1a	1121.54 ± 385.88 (898.74; 1344.34)	1007.70 ± 358.86 (800.50; 1214.90)	Group: F = 2.70; *p* = 0.11; η^2^ = 0.09 Before–After: F = 3.79; *p* = 0.01; η^2^ = 0.13 Group Before–After: F = 0.58; *p* = 0.63; η^2^ = 0.02
	1b	1377.68 ± 354.66 (1172.91; 1582.46)	1181.25 ± 447.07 (923.12; 1439.38)	
	1c	1401.98 ± 459.88 (1136.46; 1667.51)	1175.67 ± 458.77 (910.78; 1440.55)	
	1d	1501.20 ± 660.08 (1120.08; 1882.32)	1160.54 ± 414.02 (921.49; 1399.59)	
Musculotendinous Stiffness [N/m]	1a	13,605.30 ± 4392.73 (11,069.01; 16,141.59)	11,838.15 ± 4027.11 (9512.97; 14,163.34)	Group: F = 2.15; *p* = 0.16; η^2^ = 0.076 Before–After: F = 10.46; *p* < 0.0001; η^2^ = 0.29 Group Before–After: F = 0.18; *p* = 0.91; η^2^ = 0.0069
	1b	15,317.13 ± 3435.42 (13,333.57; 17,300.68)	13,211.77 ± 4789.01 (10,446.68; 15,976.86)	
	1c	16,910.75 ± 4940.17 (14,058.38; 19,763.12)	14,656.68 ± 3974.47 (12,361.88; 16,951.47)	
	1d	15,365.27 ± 3955.07 (13,081.68; 17,648.86)	12,804.93 ± 4716.03 (10,081.97; 15,527.88)	

## Data Availability

The original contributions presented in this study are included in the article. Further inquiries can be directed to the corresponding author.

## Abbreviations

The following abbreviations are used in this manuscript:

EG	Experimental Group
CG	Control Group
SJ	Squat Jump
CMJ	Countermovement Jump
slCMJ	Singe Leg Countermovement Jump
RSI	Reactive Strength Index
